# State of the Art in Dual-Curing Acrylate Systems

**DOI:** 10.3390/polym10020178

**Published:** 2018-02-12

**Authors:** Osman Konuray, Xavier Fernández-Francos, Xavier Ramis, Àngels Serra

**Affiliations:** 1 Thermodynamics Laboratory, ETSEIB, Universitat Politècnica de Catalunya, Av. Diagonal 647, 08028 Barcelona, Spain; xavier.fernandez@mmt.upc.edu (X.F.-F.); ramis@mmt.upc.edu (X.R.); 2 Department of Analytical and Organic Chemistry, Universitat Rovira i Virgili, C/Marcel·lí Domingo s/n, 43007 Tarragona, Spain; angels.serra@urv.cat

**Keywords:** acrylates, methacrylates, dual-curing, click chemistry

## Abstract

Acrylate chemistry has found widespread use in dual-curing systems over the years. Acrylates are cheap, easily handled and versatile monomers that can undergo facile chain-wise or step-wise polymerization reactions that are mostly of the “click” nature. Their dual-curing processes yield two distinct and temporally stable sets of material properties at each curing stage, thereby allowing process flexibility. The review begins with an introduction to acrylate-based click chemistries behind dual-curing systems and relevant reaction mechanisms. It then provides an overview of reaction combinations that can be encountered in these systems. It finishes with a survey of recent and breakthrough research in acrylate dual-curing materials for shape memory polymers, optical materials, photolithography, protective coatings, structured surface topologies, and holographic materials.

## 1. Introduction

A dual-curing process is defined as a combination of two curing reactions taking place simultaneously or sequentially [1]. It is a method to combine two otherwise distinct polymer networks. The resulting interpenetrating polymer network (IPN) exhibits superior properties compared to its individual parts. If a functional group participates in both curing reactions, grafted IPNs can be obtained. Sequential dual-curing facilitates ease in processing and handling as long as the intermediate materials (i.e., after the first curing reaction) are chemically stable and their properties tailorable. If the dual-curing process merely aims for improved final properties, control of the reaction sequence may be irrelevant. Sequential dual-curing methodology is developed from multi-stage (or B-stage) processing techniques. Conventionally, multi-stage processing refers to a one-pack adhesive formulation, usually of epoxide origin, that can be partially pre-cured after application to a substrate. The adhesive applied substrate can be transported and/or further processed (such as in a final assembly stage), and the final cure of adhesive could be initiated whenever desired by using heat or ultraviolet (UV) light, depending on formulation chemistry. The same technology finds use in processing of complex composite parts as well [2,3]. In contrast, two-pot systems do not feature such flexibility in processing as the final curing reaction starts once the initial components are mixed. Multi-stage processing is well known from the early times of crosslinked polymers, with the development of Bakelite in the early 20th century. Pre-polymer synthesis and their further crosslinking is a common processing technology [4,5,6]. 

**Scheme 1:**
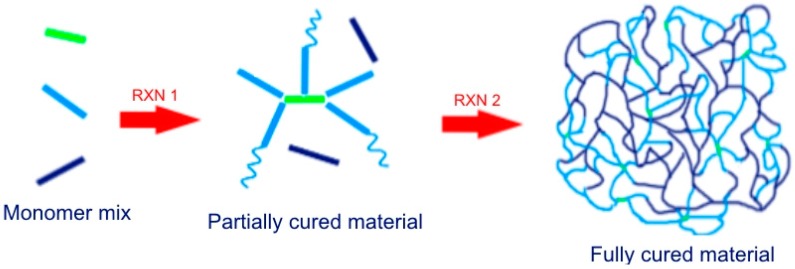
A sequential dual-curing process. The monomers depicted as green and light blue participate in the first curing reaction (RXN 1) leaving the dark blue monomers unreacted. At a later stage (RXN 2), the dark blue monomers polymerize with the available light blue functional groups and also homopolymerize. A grafted IPN is obtained.

In dual-curing processing, the extent of both curing reactions and the properties of the intermediate material can be easily controlled by changing the composition of the formulation, as well as the duration and temperature of cure [1]. In the case of sequential dual-curing, the intermediate material will have stable properties and will not cure unless the second reaction is triggered. In comparison with pre-polymer synthesis approaches, processing and compounding is much easier in dual-curing formulations. The pre-polymer is produced in-situ during the first curing stage of the initial mixture of low molecular weight monomers, rather than by mixing of larger polymeric components. This is of great use when low-viscosity formulations are required by the application. However, the concept of dual-curing goes far beyond such concepts and makes it possible to develop custom-tailored materials with a processing flexibility that is useful for a number of advanced, high-added-value applications. Dual-curing systems facilitate solvent-free processing, starting from liquid monomers, and mild processing conditions, which helps minimize the emission of volatile organic compounds (VOCs) to the atmosphere and reduce processing energy costs. An exemplary sequential dual-curing process is schematized in Scheme 1. 

Successful sequential dual-curing processing requires that (a) both polymerization reactions are selective and compatible so that no undesired inhibition or reactivity effects take place; and (b) they can be triggered using different stimulus such as UV light or temperature, or else they have sufficiently different reaction rates so that they can be controlled from a kinetics point of view in terms of time and temperature. An important advantage of sequential dual-curing is that the properties of the final and intermediate stages can be custom-tailored, which is of key importance for their use in advanced applications. 

The control of the curing sequence may not be relevant if such combination aims only at the enhancement of the properties of a given base material. For instance, post-polymerization of excess groups in off-stoichiometric step-wise curing systems is used to enhance the thermal–mechanical properties of thermosets [7,8]. In other cases, the crosslinking reaction is triggered by means of UV light leading to fast cure in the outermost layers, but extended through-cure can take place more slowly and reach completion by means of a thermal polymerization process in the deepest layers [9]. Likewise, for certain applications such as coatings that are cured by UV light, the use of dual-curing formulations together with the use of photoabsorbers can make it possible to achieve complete, or at least sufficient, cure in shadowed areas [10]. In the synthesis of IPNs [11,12,13,14,15], the control of the dual-curing process plays a role in the morphology. Materials with particular morphological features can be developed depending on whether the polymerization processes take place in a simultaneous or sequential way, and whether or not there is crosslinking between the two networks. 

Dual curable systems are also available in which the two curing reactions proceed simultaneously. In such systems, the ultimate goal of employing a dual-cure formulation is enhancing end-product properties rather than an intermediate processing possibility. For example, mixtures of multifunctional acrylate and epoxy monomers can be crosslinked simultaneously under UV light and with the use of radical and cationic photoinitiators. Decker and coworkers [16] showed the synthesis of IPNs from a 50:50 mixture (by weight) of 1,6-hexanediol diacrylate (HDDA) and epoxidized soybean oil (ESO) under intense UV irradiation. The plasticizing effect of ESO facilitated higher conversion of HDDA compared to a neat formulation. Such acrylate/epoxide IPNs are suitable as fast-drying protective coatings, composites and optical components. 

The use of photolatent and thermally-latent catalysts, for base- or acid-catalysed reactions or radical polymerization reactions [17,18,19] can contribute to the design of efficient one-pot dual-curing formulations. These formulations have good storage stability of the uncured system and stable intermediate properties. The use of blocked species can also be exploited for the design of dual-curing formulations [20]. Careful control of the exothermicity of polymerization is a general concern in composites processing for control and quality purposes [3,21]. It is also a factor to be taken into consideration in dual-curing processing schemes, especially in the case where the second polymerization process is thermally activated [22].

The intermediate and final properties of dual-curing materials strongly depend on the network structure at the end of each polymerization process. Some criteria for dual-curing formulation design or preliminary network structure analysis based on ideal network build-up models have been outlined recently [23,24]. However, recent studies [25] suggest the need for a deeper understanding of the effect of monomer feed ratio and monomer structure and functionality on the structure-property relationships in dual-cure processing. 

A large number of reactions can be used in dual-curing thermosetting formulations [1] but “click” type reactions are of special interest. The click concept was first coined by Sharpless et al. to refer to a variety of chemical reactions that are orthogonal, selective, and highly efficient [26,27]. Click reactions have the following characteristics: (a) high yields; (b) regio and stereospecificity; (c) insensitivity to oxygen or water; (d) mild, solvent-less, or aqueous reaction conditions; (e) orthogonality with other common organic reactions; and (f) applicability to a wide range of available starting compounds [28,29]. The click chemistry concept has found a particular interest in polymer chemistry leading to a “paradigm change”: A modular construction approach is used [27,30,31] rather than conventional synthetic approaches. Product purification is mostly unnecessary thanks to the selectivity and quantitative yield of click reactions. In an extensive review, Tunca reported the combination of two or more orthogonal click processes to synthesize complex architectures [32]. 

**Scheme 2:**
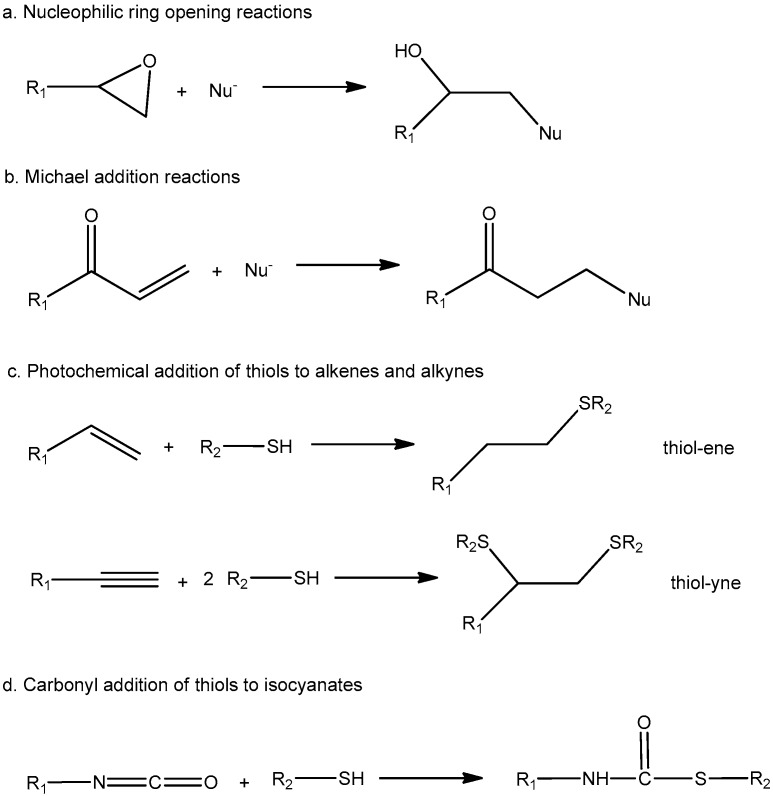
Reaction schemes of several click reactions.

In the field of thermosets, click reactions allow the preparation of well-defined network structures. They can be carried out under mild and solvent-free conditions and therefore can be designed in an environmentally friendly manner. As various chemical functionalities show click behaviour and each has chemically and mechanistically distinct reaction pathways, they can be combined in dual curing methodologies. Some common click reactions reported for the preparation of dual-curable materials are outlined in Scheme 2.

## 2. Acrylate Reactions in Dual-Curing Systems

Acrylic monomers have been used widely in polymer formulations since decades. There is a vast selection of commercially available, affordable and easily handled acrylates. By carefully selecting acrylate monomer structure and the functionality, polymers can be synthesized for a plethora of industrial applications such as industrial coatings [13,33,34], adhesives [35,36], inks [37], and biomaterials [38]. Acrylate groups readily react with a variety of substrates owing to the carbon–carbon double bond and its neighbouring electron withdrawing carbonyl group, which enhances the reactivity of the double bond. Therefore, they are widely used in a number of reactions [39,40,41,42,43,44] that can fit the click concept. 

A prominent click reaction of acrylates is the Michael addition. Michael addition [39] is defined as the 1,4-addition (or conjugate addition) of resonance-stabilized carbanions to activated double bonds. Michael-type addition reactions are used widely in dual curing processes, because of the variety of commercially available nucleophiles (Michael donors) and activated double bond compounds (Michael acceptors) that can be used in such processes. 

Typical Michael donors are amines, thiols and acetoacetates. The group of Michael acceptors is more numerous and includes acrylate esters, acrylamides, maleimides, alkyl methacrylates, cyanoacrylates and vinyl sulfones. Of all these different acceptors, acrylates have a suitable combination of reactivity, versatility, availability and cost. Although there are Michael acceptors with a higher reactivity, such as vinyl sulfones [24], there is a vast selection of commercially available and affordable acrylates. In contrast, widely available methacrylates have much lower reactivity due to the steric effect of the methyl group [45] and are therefore less used as Michael acceptors.

As co-reactant in Michael additions, the most common nucleophylic monomers are thiols (thiol–Michael addition), amines (aza-Michael addition), and acetoacetates. As these polymerization routes of acrylates usually fit click criteria, they are used widely in dual-curable formulations, in combination with other click reactions, or with other non-click reactions. 

A variety of basic and, especially, nucleophilic catalysts can be used to trigger and control Michael additions [39,46]. Michael-type (mostly thiol–Michael) reactions can be combined in dual-curing systems with a variety of other reactions such as radical-mediated polymerizations in a controlled and sequential way. However, the complex dependence of thiol–Michael additions on the solvent, nature and concentration of catalyst, polarity and basic/nucleophilic character of the catalyst makes it difficult to optimize the kinetics and extension of the reaction and to minimize side reactions [47]. The prediction of the optimum reaction conditions based on the pK_a_ (negative logarithm of the acid dissociation constant) of thiols and bases is not possible due to the concurrence of base and nucleophile mediated thiol–Michael reactions [46]. The use of strong bases or nucleophiles leads to a very fast reaction, but makes preparation difficult [24,46]. Weak nucleophiles allow the control of the initial reaction rate, showing an induction period that can be further tuned by addition of small quantities of an acid (protonic species) [48]. The anionic polymerization of acrylates, which relies upon Michael addition chemistry, is another side reaction that cannot be disregarded taking place during the curing of thiol/acrylate systems [39]. Photobase generators can be used in Michael addition reactions to design latent dual-curing systems [45,49,50] but a radical inhibitor is needed to suppress the radical mediated side reactions [49,50,51]. 

Radical-mediated reactions such as acrylate homopolymerization or thiol–ene reaction can be found in dual-curing systems [1]. These reactions, although not fitting the click concept, are also highly versatile and can benefit from selective activation by means of a number of thermal initiators or photoinitiators [52,53,54,55,56,57].

### 2.1. Thiol–Acrylate Reaction

**Scheme 3:**
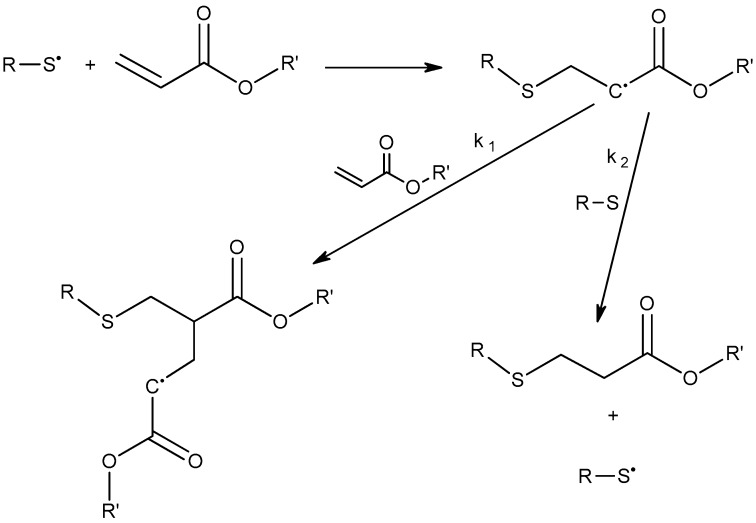
Radical-mediated thiol–acrylate polymerization. The propagation kinetic constant (*k*_1_) was reported to be 1.5 higher than the hydrogen abstraction kinetic constant (*k*_2_) [58].

The most common and clickable co-reactants for acrylates are thiols. Acrylates react with thiols via two distinct mechanisms: (a) Radical mediated thiol–acrylate reaction; (b) Thiol–acrylate Michael addition. The radical mediated route is shown in Scheme 3. The thiyl radical is formed either by UV irradiation in the presence of photoinitiators, or by thermal activation [52]. Once the radical is generated, it is added to the double bond of theacrylate monomer to yield an acrylic radical. In turn, this radical specie either propagates through reaction with another acrylate, or abstracts a hydrogen from the thiol to yield the thiol–acrylate reaction product. In polymeric systems, a competition exists between the chain-growth homopolymerization of the acrylate and the step-growth mechanism of a thiol–ene polymerization. It has been shown previously by Cramer and Bowman [58] that the rate constant for acrylate propagation is 1.5 times higher than that of hydrogen abstraction from the thiol. Because of this, a radical mediated thiol–acrylate reaction cannot be considered click since thiols remain partially unreacted. 

**Scheme 4:**
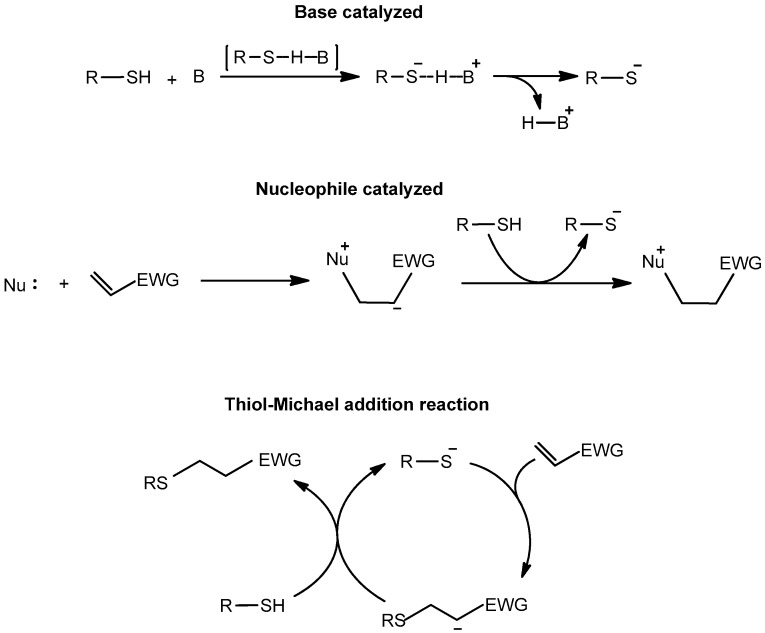
Thiol–Michael reaction mechanism. Thiolate anion may form via two distinct mechanisms: Catalysis by base or nucleophile. (EWG represents an electron withdrawing group.)

The thiol–acrylate Michael addition (thiol–Michael in short) reaction starts with the formation of a thiolate anion, which could take place via two different mechanisms, as shown in Scheme 4 [59].

Depending on the p*K*_a_ and nucleophilic strength of the catalyst, the formation of the thiolate can follow either mechanism. When a strong base such as 1,5,7-triazabicyclo[4.4.0]dec-5-ene (TBD) is used, the base catalysed mechanism is favoured for the thiol–Michael reaction. On the other hand, the reaction follows the nucleophilic path when strong nucleophiles are used as catalyst such as 4-(*N*,*N*-dimethylamino)pyridine (DMAP) [60,61] or tertiary phosphines [46]. Thiol–Michael reactions generally fit click criteria as the reaction can be carried out in an eco-friendly manner. There are no competing reactions within the mechanism, thereby allowing quantitative conversions of both reactants. As a result, this reaction has been combined with many other click reactions to develop dual-curing systems over the years. 

### 2.2. Aza-Michael Addition

**Scheme 5:**
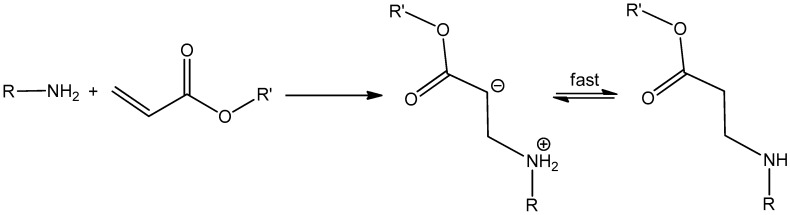
Aza-Michael addition between a primary amine and an acrylate. The resulting secondary amine may further undergo aza-Michael addition at a slower reaction rate.

Acrylates can also undergo Michael addition in the presence of amines. The so-called aza-Michael additions have the same mechanism as thiol–Michael reactions (Scheme 5). Amines as Michael donors have the following advantages in comparison to thiols or acetoacetates: (a) many suitable amines are commercially available, including amine-terminated hyperbranched polymers; (b) catalysts are not required since amines can act as both nucleophiles and bases; (c) radicals or other active species are not formed during the first stage of curing; (d) the presence of tertiary amines formed during aza-Michael addition overcomes the intrinsic oxygen inhibition of acrylate free-radical polymerizations [62] and allows the curing to be performed in non-inert environments and (e) these tertiary amines can act as co-initiator when type II photoinitiators (i.e., photoinitiators that require a co-initiator or synergist to produce initiating radicals) are used. Due to the low reactivity of formed secondary amines, aza-Michael reaction may not reach completion but further crosslinking during subsequent photoirradiation leads to complete acrylate conversion [42].

### 2.3. Acetoacetate–Acrylate Reaction

**Scheme 6:**
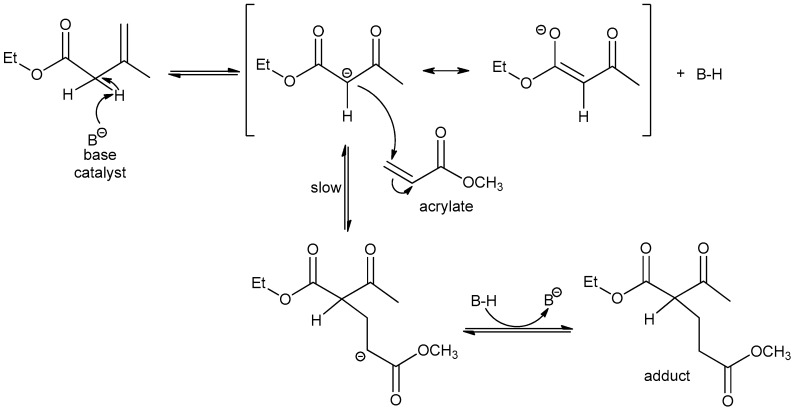
Michael reaction mechanism. The Michael donor is deprotonated by the base catalyst. The formed anion attacks the acrylate double bond and regenerates the base. The adduct formed may further undergo a second but slower Michael addition.

Acetoacetate–acrylate reaction [39] is a good example of Michael addition to acrylates. Mechanistically, the reaction proceeds through an enolate anion, which is formed after the deprotonation of the active methylene. The deprotonation is facilitated by the base catalyst (Scheme 6). The enolate then adds to the acrylic double bond. The adduct is formed and the base is regenerated once proton transfer occurs. The rate determining step of the reaction is the attack of the enolate to the acrylate. The reaction kinetics is highly dependent on base strength. The formed adduct may undergo a second Michael addition, but at a slower rate. 

### 2.4. Radical Homopolymerization of Acrylates

**Scheme 7:**
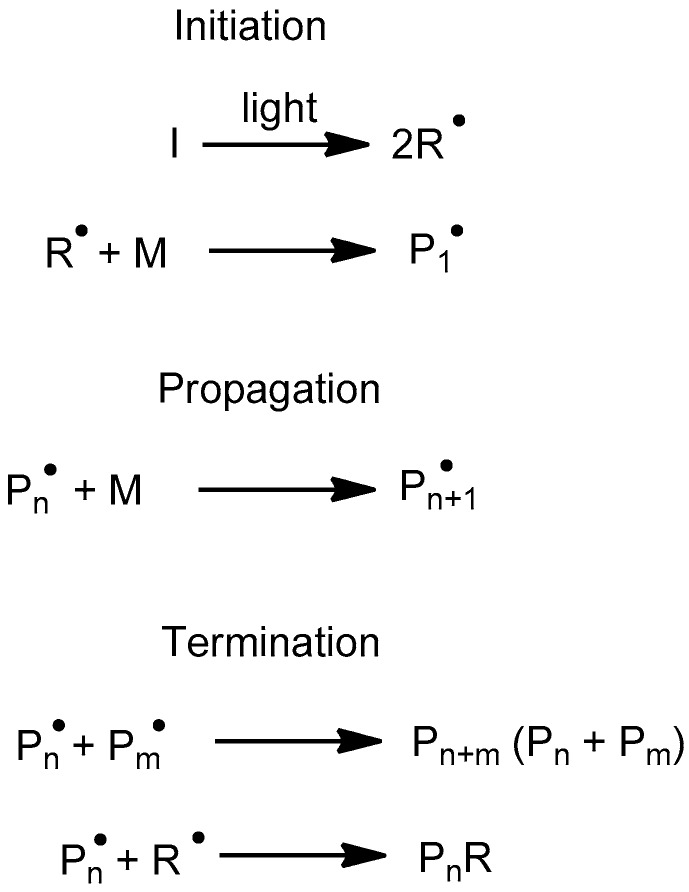
Radical mediated photopolymerization of acrylates. I: Photoinitiator, R: Radical, M: Monomer, P: Polymer chain.

For the sake of completeness, we present the well-known radical mediated photopolymerization reaction of acrylates in Scheme 7. The reaction starts with the photolysis of the initiator that yields two equally reactive radicals. The next step is the initiation of the chain by the reaction of a radical with an acrylate monomer. The chain propagates by reaction with other monomers. Termination occurs when the propagating polymer reacts either with another growing polymeric radical or with a primary radical. 

## 3. Acrylate-Based Dual-Curing Systems

### 3.1. Thiol–Acrylate and Thiol–Epoxy Reactions

Although the radical-mediated thiol–acrylate reaction does not generally exhibit click characteristics, it has been combined with other click reactions to obtain materials with significantly low soluble content. Jian et al. [50] obtained hybrid organic networks by thiol–epoxy/thiol–acrylate polymerization processes in which both reactions proceeded sequentially without clear separation. The curing was triggered by irradiation with UV light by the use of a photobase generator 1,5,7-triazabicyclo[4.4.0]dec-5-enyl tetraphenylborate (TBD·HBPh_4_) capable of in situ generating a strong base and free radicals, with isopropylthiolxanthone (ITX) as photosensitizer to extend the wavelength absorption range of the catalyst. The free radicals facilitated both thiol–acrylate radical polymerization as well as acrylate homopolymerization, leading to materials with unreacted thiol groups. However, as the photobase generator also liberated a strong base (TBD), thiol–Michael addition also took place and compensated partly for the stoichiometric imbalance. The researchers found that thiol–acrylate reactions and acrylate homopolymerization were faster and more efficient than the thiol–epoxy reactions (>95% conversion in a matter of seconds and minutes, respectively), and the thiol conversion increased with an increase in the epoxy content. 

In a recent paper [61], the authors of this review presented a novel photolatent dual-cure thiol–acrylate–epoxy system. Under UV light, the photobase generator liberates a strong base that catalyses the thiol–Michael reaction at room temperature. To initiate the second curing reaction between epoxy and remaining thiols, the system is heated above 80 °C. The use of a radical inhibitor ensured that no radical mediated acrylate homopolymerization took place. The final material properties suggested potential in application such as chemically resistant industrial coatings, adhesives, and electronic materials. 

It is also reported in the literature the preparation of hybrid polymer networks (HPNs) from one-pot thiol–acrylate–epoxy mixtures. Jin et al. [63] cured a stoichiometric combination of a diacrylate and a diepoxide using difunctional or multifunctional thiol crosslinkers. The nucleophilic thiol–acrylate reaction and thiol–epoxy reaction was carried out simultaneously at 80 °C in the presence of 1,8-diazabicyclo[5,4,0]undec-7-ene (DBU). Depending on the functionality of the thiol, resulting materials exhibited a wide range of properties characterized by differential scanning calorimetry (DSC), dynamic mechanical analysis (DMA) and scanning electron microscopy (SEM) methods. 

### 3.2. Thiol–Acrylate and Thiol–Ene (or Thiol–Yne) Reactions

Chatani et al. [64] used selective and sequential thiol–divinyl sulfone and thiol–Michael additions to create materials with independent network structures and two different glass transition temperatures showing multiple shape-memory effect. Both reactions were catalysed by nucleophilic phosphine catalysts. It was possible to separate the process into two reaction steps since the two thiol monomers used (a mercaptoacetoacetate and a mercaptopropionate) showed significantly different reactivities. 

Chan et al. [65] showed the possibility of combining phosphine-catalysed Michael addition of thiols to acrylates followed by UV-induced radical thiol–yne addition in a controlled and sequential way.

Thiol–Michael addition to acrylates and thiol–ene click reactions were later combined by Peng et al. [66] in a sequential way using a tertiary amine for the thiol–Michael addition and a radical photoinitiator for the thiol–ene reaction for the preparation of holograms. A similar approach was used for sequential thiol–Michael and thiol–yne systems [67].

Recently, a thiol–acrylate and thiol–ene dual-curing system was developed [68]. First stage of curing is a click thiol–Michael reaction catalysed by a novel set of tertiary amines comonomers with allyl functionality. These allyl moieties later undergo a radical thiol–ene reaction to yield fully crosslinked dual-networks. 

### 3.3. Thiol–Acrylate and Acetoacetate–Acrylate Reactions

Using acetoacetates to accompany thiols as Michael donors is a strategy to achieve sequentiality in dual-curing processes since the proton acidities of acetoacetate and thiol groups are disparate. Recently, our group [43] developed dual-curable thiol–acetoacetate–acrylate thermosets by using thiol–Michael addition to acrylates at room temperature followed by Michael addition of acetoacetates to acrylates at moderately elevated temperature. The different acidities of the protons on thiol and acetoacetate groups, and the favorable p*K*_a_ of the base catalyst ensured two separate curing stages. Using a photobase generator, it was also possible to design latent curing systems out of the same mixtures. Similarly to other dual-curing systems, the final material properties could be tailored by changing the structure of the monomers and the relative contribution of both Michael addition reactions, by only changing the monomer proportion.

### 3.4. Thiol–Acrylate and Thiol–Isocyanate Reactions

Thiol–isocyanate coupling and thiol–Michael addition reactions were combined by Matsushima et al. [69] to synthesize materials with tunable mechanical properties. Thiolisocyanate co-catalysed by phosphine–acrylate was faster than the thiol–Michael addition at low-phosphine concentration. Therefore, the curing steps were easily separable. Although phosphines were not basic enough to initiate a thiol–isocyanate reaction, the phosphonium enolate generated via nucleophilic addition of the phosphine to acrylate double bond acts as a strong base that can deprotonate the thiol and produce the thiolate anion, which can then react with isocyanate groups. In the presence of excess acrylate groups, a final photopolymerization step could be used to produce ternary networks.

### 3.5. Combination of Two Thiol–Michael Additions on Different Acceptors

Dual curing systems can be designed based on a single reaction mechanism, but using monomers whose reaction kinetics have different temperature dependencies so that the two stages can be carried out at different curing temperatures. As an example, thiol–Michael addition was employed in a sequential curing process using two monomers with distinct electrophilic strengths: vinyl sulfones and acrylates [64]. The former reacted at room temperature, whereas the latter reacted at 90 °C.

Acrylates are more reactive than methacrylates in Michael additions due to the inductive effect and steric hindrance of the methyl group in the methacrylate. Thiol–Michael reactions with acrylates and methacrylates have been be employed in dual curing formulations by choosing an adequate combination of catalysts: A conventional tertiary amine for the thiol–Michael addition to the acrylate in dark conditions and a photolatent base leading to the release of a stronger base for the thiol–Michael addition to the methacrylate [45]. These formulations were used in photo-patterning with spatial and temporal control. 

### 3.6. Michael Addition Followed by Homopolymerization

In order to obtain storable and processable polymers at the final of the first stage of curing and final networks with optimum properties for different applications, the preparation of dual materials from off-stoichiometric formulations has recently gained interest [59]. The monomer in excess should be able to undergo homopolymerization reaction, without any interference. The first stage is a self-limiting click reaction between two multifunctional monomers with an excess of one of the two monomers. The second stage reaction is a thermally or UV-induced polymerization of the excess of unreacted groups. The materials obtained after the first curing stage can be gelled or ungelled and loosely or tightly crosslinked at the end of the second curing stage, depending on the functionality and feed ratio of the monomers.

The combination of step-growth (usually first stage) and chain-growth (usually second stage) polymerization reactions confers these systems a great versatility, and complete curing can be generally achieved at the end of the second curing step. Crosslinked polymer networks obtained during the first stage of curing via step-growth polymerization reactions have some remarkable features such as homogeneity, high capacity for mechanical energy absorption near its glass transition temperature, high gel point conversion and low polymerization-induced shrinkage stress compared to chain-growth polymers. In applications that require high *T*_g_ value and hardness at ambient temperature, the relatively low *T*_g_ values and crosslinking densities of the step-growth intermediate polymers can increase significantly after the second stage of curing via chain-growth homopolymerization. 

As Michael addition reactions are self-limiting, all Michael donor species will have reacted by the end of the first stage of a dual-curing scheme. The remaining Michael acceptor species, usually acrylates, can homopolymerize in the second curing stage.

Moszner et al. [70,71] synthesized networks from multifunctional acetoacetates (as Michael donors) with multiacrylates (as Michael acceptors) using 1,5-diazabicyclo-[4.3.0]non-5-ene (DBN) as the base catalyst. Tailor made materials could be prepared in a two-step process: Michael addition followed by radiation curing of excess acrylate. An analysis of crosslinked polymers showed that the longer spacer between β-ketoester groups and the lower functionality and higher flexibility of the reaction components made it possible to reach higher degrees of conversion. The vinyl monomers in excess, which act as reactive solvent in the first curing stage, had a positive effect on the homogeneity, conversion and crosslinking density of the synthesized networks. During the second stage, the soft Michael network was converted into a hard crosslinked material with high Shore hardness. The prepared materials were interpenetrating polymer networks covalently bonded. To synthesize similar materials, Moy et al. [72] used Michael addition of acetoacetates to multi-acrylate followed by photopolymerization of acrylates that were in excess in the initial mixture. The Michael addition has been used to synthesize liquid oligomers that find use in UV-curable formulations [73]. This alleviates the need for toxic photoinitiators, which would be indispensable in conventional solvent-based formulations to ensure the complete cure of thick films [37]. 

Despite the promising results obtained by Moszner et al., few studies have deepened in the formation of networks based on acetoacetates and acrylates. Probably, the relatively high complexity of synthesized acetoacetates of different functionalities and structure along with the relatively lower reactivity of the second acidic proton in the methylene of the acetoacetate are the reasons for the scarce number of publications on this specific type of Michael addition [74].

Taking advantage of the behaviour associated with click reactions, Bowman’s group [41,75,76] has developed a novel two-stage reactive system using off-stoichiometric thiol–acrylate formulations, in a similar way to the one developed by Moszner et al. [70,71] with acetoacetate–acrylate formulations. In the first step, a polymer network is formed via base-catalysed thiol–Michael addition. In the second step, the excess of acrylate groups undergo photoinitiated free radical polymerization. Since acrylate groups react in both stages of curing, this ensures covalent bonding between poly(thio-ether) and poly(acrylate)s networks, rendering the final materials suitable for certain applications in which extractable material is not desirable. The strategy of combining a click reaction with homopolymerization yields two distinct and independent sets of material properties at the end of both stages. In general, after the first stage of curing, the materials can be stored, shaped and processed. Later, their properties can be dramatically increased through the second curing stage. By controlling the monomer ratio in the formulation and the functionality and rigidity of the monomers, materials with a broad range of applications, such as shape memory polymers and imprinting and optical materials can be obtained, as will be later shown. 

**Scheme 8:**
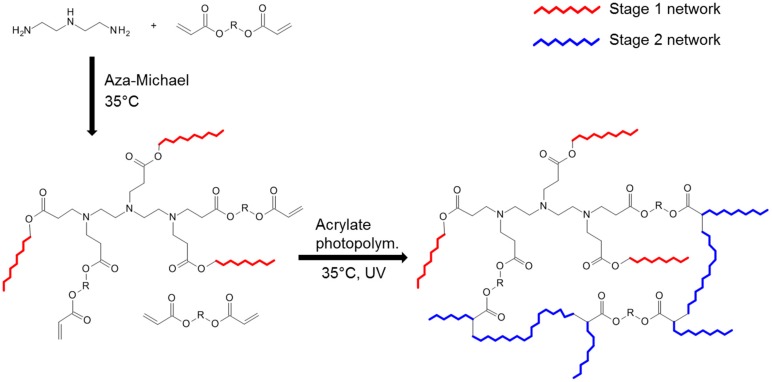
Aza–Michael reaction followed by acrylate homopolymerization under UV light. Both reactions were carried out at room temperature. Stage 1 network contains unreacted acrylate groups that photopolymerize later in Stage 2.

The replacement of thiols or acetoacetates by amines as Michael donors has been recently explored as a way to prepare a new family of poly(amino ester)-*co*-poly(acrylate) thermosets based on off-stoichiometric amine-acrylate formulations [42], in a dual-curing solvent-free process at room temperature. The first stage of curing is a self-limiting aza–Michael addition between amine and acrylate groups and the second stage is a radical photopolymerization of the acrylates in excess. The dual-curing procedure is shown in Scheme 8.

Based on the same idea, Retailleau et al. [40] took advantage of the different reactivities of primary and secondary amine groups to synthesize polymeric networks via three time-controlled steps using double click Michael addition and radical photopolymerization.

### 3.7. Michael Addition Followed by Methacrylate Homopolymerization

**Scheme 9:**
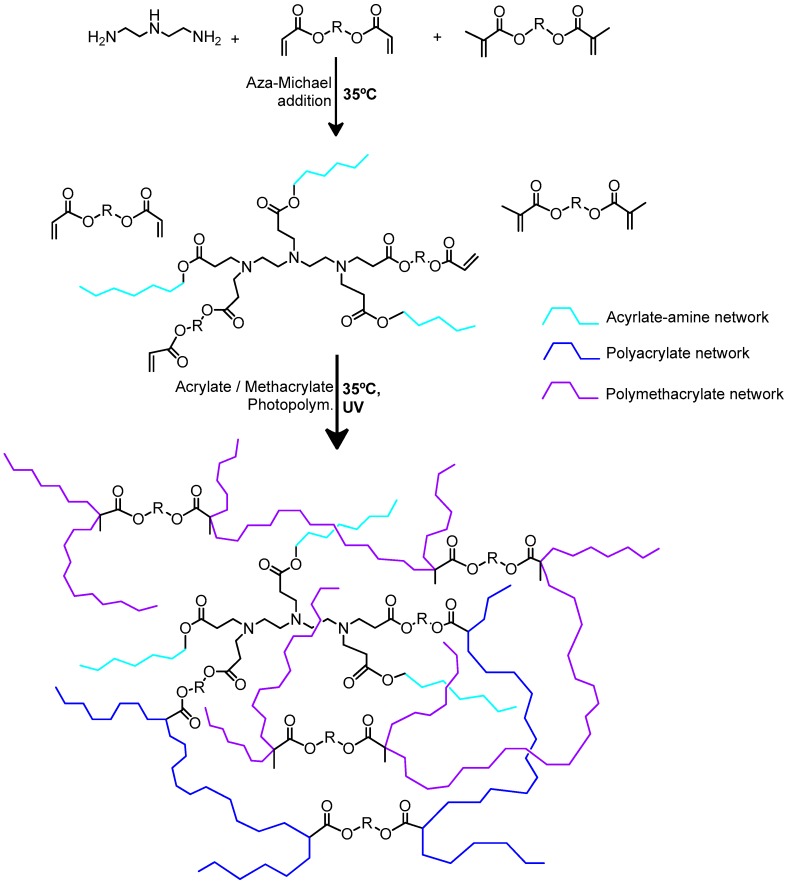
A room temperature dual-curing process for an amine–acrylate–methacrylate. Stage 1 (acrylate–amine) network contains unreacted acrylate and methacrylate groups. UV curing at the final stage ensures all these unreacted acrylate or methacrylate groups to photopolymerize.

Methacrylates are less reactive in Michael additions than acrylates due to the inductive effect of the methyl group in the methacrylate and to the steric hindrance caused by the methyl in *α* position [39,45]. Accordingly, in the presence of acrylates, methacrylate groups remain practically unreacted after Michael addition and participate preferably in the homopolymerization process. Recently, the authors of this review [44] have presented an efficient dual curing scheme for a set of mixtures containing different proportions of acrylates and methacrylates (Scheme 9). The first curing stage is a stoichiometric aza–Michael addition between acrylates and an amine, followed by photo-initiated radical homopolymerization of methacrylates and remaining acrylates. An analysis of aza–Michael reaction kinetics confirmed that amines react selectively with acrylates, leaving methacrylates unreacted after the first curing stage. Depending on the weight percentage of the constituent polymeric networks, a wide range of final material properties can result. 

With a similar approach, Klee and co-workers [77] employed aza–Michael addition of diamines to acryloxylethyl methacrylates to synthesize crosslinkable oligomers for dental composites that exhibited low curing shrinkage upon photocuring.

In a similar manner, dual-curing acetoacetate-acrylate/methacrylate formulations have been prepared and characterized by our research team [78]. The first stage of curing was a base-catalysed Michael addition between acetoacetate and acrylate groups and the second stage was methacrylate radical photopolymerization. Both are ambient temperature reactions. Because methacrylates are poor Michael acceptors, they did not react with the acetoacetate, thereby ensuring staged curing. The materials gelled after the Michael addition and the final properties could be tuned by changing the relative contribution of the Michael and radical homopolymerization reactions. The latency of the formulations at the beginning of the second stage can be regulated as desired by UV irradiation.

### 3.8. Isocyanate–Thiol Followed by Acrylate Homopolymerization

**Scheme 10:**
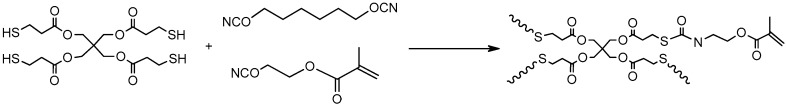
Thiol–isocyanate click reaction using a monomer that wields both isocyanate and methacrylate functionalities. The resulting first-stage network can be further crosslinked at a later stage using photo-induced methacrylate homopolymerization. Scheme adapted from [79].

There are other choices for first stage reactions followed by curing of homopolymerizable groups. Podgórski et al. [79] employed a base-catalysed thiol–isocyanate click coupling with free radical methacrylate homopolymerization in a two-stage process for the fabrication of well-defined surface patterns as well as functional geometric shapes. The second stage materials were obtained upon UV irradiation and using a photoinitiator, and a photoabsorber. They used a monomer with both isocyanate and methacrylate functionality that was capable of participating in the thiol–isocyanate reaction (Scheme 10) and was able to further crosslink at the second curing stage. 

### 3.9. Diels–Alder Reaction Followed by Acrylate Homopolymerization

The Diels–Alder reaction, which also has a click nature, was recently combined with UV-induced acrylate homopolymerization by Bowman and co-workers [80]. From mixtures of bismaleimides, acrylates and monomers with furane moieties, new thermosets by dual curing were prepared. The contribution of the Diels–Alder reaction to the network structure depended on the temperature-dependent equilibrium, because of the reversible character of this reaction. Unreacted maleimide moieties could become incorporated into the acrylate network while the resulting material could maintain its thermoreversible behaviour thanks to the underlying Diels–Alder network. 

### 3.10. Combination of Acrylate Homopolymerization and Other Reactions

Nowers and co-workers [11] have investigated IPNs synthesized from a combination of radical mediated acrylate homopolymerization and cationic epoxy homopolymerization. They explored the impact of composition and reaction sequence on conversion, rheological and mechanical properties. The structure-property relationships they discovered were nonlinear and complex. They found out that maximum conversions could be achieved at intermediate compositions due to the dilution effect. Elastic modulus increased with increasing epoxy content, whereas yield strain increased with increasing acrylate content. Interestingly, they found out that the reaction sequence had a significant effect on final glass transition temperatures: Higher glass transition temperatures (*T*_g_) were obtained if epoxies were homopolymerized before acrylates. 

Cationic epoxy polymerization has been combined with radical mediated (meth)acrylate homopolymerization also by Li et al. [81] to yield IPNs for 3D printing applications. In their work, they used mixtures of difunctional (meth)acrylates and epoxides. Firstly, they irradiated the mixtures with UV light until full (meth)acrylate conversion and cationic initiation. Later, they investigated the effect of the dark curing time of the epoxy on the final properties. Interestingly, they found that the dark curing time had a significant impact on the IPNs formed. Furthermore, the extent of this variation was comparable to that of changing monomer compositions. 

**Scheme 11:**
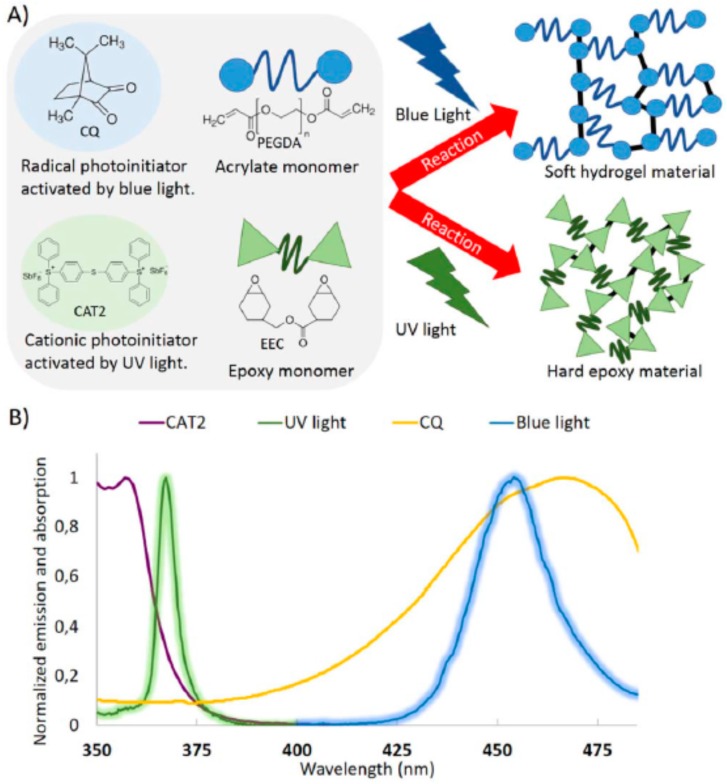
(**A**) Reaction schemes for photoinitiation of radical mediated acrylate homopolymerization and cationic epoxy homopolymerization. (**B**) Normalized emission spectra of the light sources used for initiating the reactions. Reprinted (adapted) with permission from [82]. Copyright 2016 John Wiley and Sons.

In another interesting paper, Larsen and co-workers [82] study an acrylate/epoxy dual system that can yield either a soft (acrylate based) or a hard (epoxy based) material depending on the emission range of the light used to irradiate the monomers. The compression modulus of end materials can be tailored between <100 kPa and >20 MPa. The curing process is summarized in Scheme 11.

Anionic epoxy curing was also combined with acrylate homopolymerization to design adhesive formulations [83]. Both UV and thermal curing was employed and the resulting materials were characterized with respect to adhesive strength and pendulum hardness. Both properties were found to increase with increasing epoxy content.

Polyurethane chemistry also found use in dual networks based on acrylates. Bertoldo and co-workers [84] reported the thermally induced polyurethane formation via the reaction of propoxylated aromatic epoxy diacrylated oligomer and allophanate modified polyisocyanurate, followed by photoinitiated acrylate homopolymerization to produce nanocomposite laminates. They found out that final acrylate conversion was an increasing function of thermal pre-treatment temperature. 

## 4. The Comonomer Approach

In some dual-curing formulations, certain click reactions are used to produce in situ comonomers that later catalyse the curing reaction. Starting from an initial mixture of amines, acrylates and thiols, Bounds et al. [85] prepared microfluidic devices using a cost-effective and eco-friendly procedure. Firstly, the comonomer is synthesized in situ via aza–Michael addition of a diethylamine to pentaerithritol triacrylate. The formed tertiary amines in turn catalyse thiol–Michael addition of trimethylolpropane tris(3-mercaptopropionate) to the remaining acrylate groups. The resulting materials exhibited diverse and tunable properties. 

**Scheme 12:**
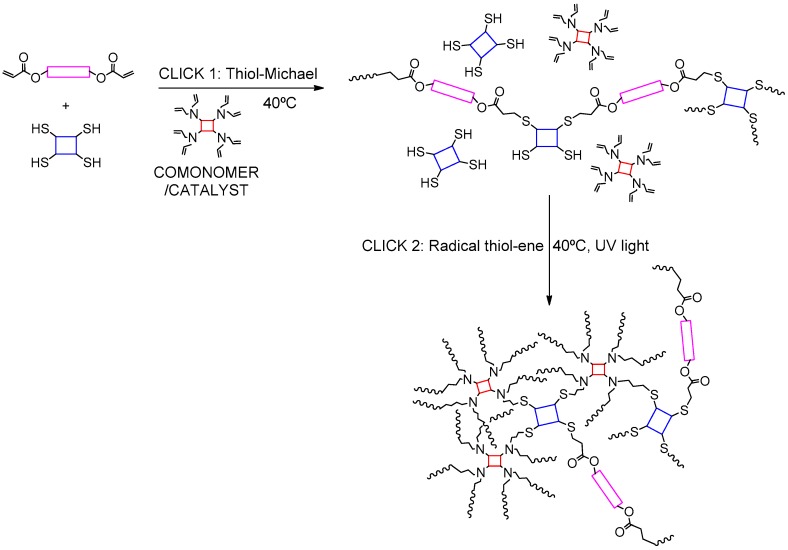
Thiol–Michael reaction catalysed by comonomer with allyl functionality. At the second curing stage, all allyl moieties get incorporated into the final network via thiol–ene radical reaction.

In a recent study by the authors of this review [68], a similar comonomer approach was employed to synthesize soft poly(thioether) thermosets using a two-step click curing procedure at room temperature (Scheme 12). The first stage of curing is a thiol–Michael addition between acrylates (or methacrylates) and thiols in excess, catalyzed by a set of pre-synthesized tertiary amine catalyst comonomers with allyl functionality. Subsequently, the pendant allyl groups of the comonomer get incorporated into the polymer network by undergoing thiol–ene UV photopolymerization with the remaining thiols. The strong catalysis by the high concentration of tertiary amine groups facilitated quantitative conversions at the end of the first stage. All remaining functional groups are depleted after a short period of UV irradiation. Final materials were clear, transparent, and they exhibited homogeneous network structures with tunable viscoelastic properties.

## 5. Polymer Modification/Functionalization

Combination of controlled/living polymerization methods and thiol–Michael reactions has also been employed to synthesize highly advanced polymer architectures such as branched or star macromolecules [59]. As the thiol–Michael reaction exhibits click characteristics, it has been combined with techniques such as reversible addition-fragmentation chain transfer (RAFT) polymerization, cobalt-catalysed chain transfer (CCT), atom transfer radical (ATRP), or ring-opening metathesis (ROMP) polymerizations. Usually, the thiol–Michael reaction is used as a post-polymerization stage to modify/functionalize the polymer. Readers are directed to [59] for a review of related systems. 

## 6. Organic–Inorganic Hybrid Dual-Curing Systems

Dual-cured processes have been proposed for the synthesis of organic–inorganic hybrid materials [86]. In that paper, the author presents a thorough review of dual-cure hybrid materials developed for interesting applications such cultural heritage protection, hydrophobic or oil-repellent coatings for textiles, dental composites, and light-emitting coatings. 

The usual method to prepare organic–inorganic hybrid systems is a photoinitiated polymerization followed by a thermal treatment for promoting sol-gel reactions of suitable alkoxyde precursors already embedded in the UV curable system. In doing so, it is possible to exploit the advantages of this fast and solvent-free radiation-induced polymerization, with the sol-gel benefits, leading to the formation of hybrid networks, generally in the form of thin coatings with superior scratch endurance, improved abrasion, heat and radiation resistance, and desirable mechanical and optical properties [87,88,89]. Vinylether, epoxide, or acrylate groups have been used as organic monomers, since they can undergo fast photopolymerizations [90,91,92] but even UV-mediated click reactions has also been applied [93,94]. Organic and inorganic precursors should be compatible in the reactive mixture. The characteristics of the final material depend on the formulation (type and proportions of organic monomers, initiator, inorganic precursors, amount of water for sol-gel process, and coupling agents) and on pH (which affects the microstructure of the inorganic phase), and time of irradiation and schedule of thermal treatment.

## 7. Applications

Acrylate dual-curing systems are advantageous as they enable two distinct and largely independent sets of material properties at each curing stages. It is possible to have a stable polymer network with desirable physical properties while maintaining the capacity to react and achieve, once initiated with an appropriate stimulus, a second and final network with enhanced material properties.

Various applications are described in the literature where acrylate dual-curing reactions are elegantly employed. The most prominent of these applications are shape memory polymers, optical materials, photolithography, protective coatings, structured surface topologies, and holographic materials.

### 7.1. Shape-Memory Systems

**Figure 1:**
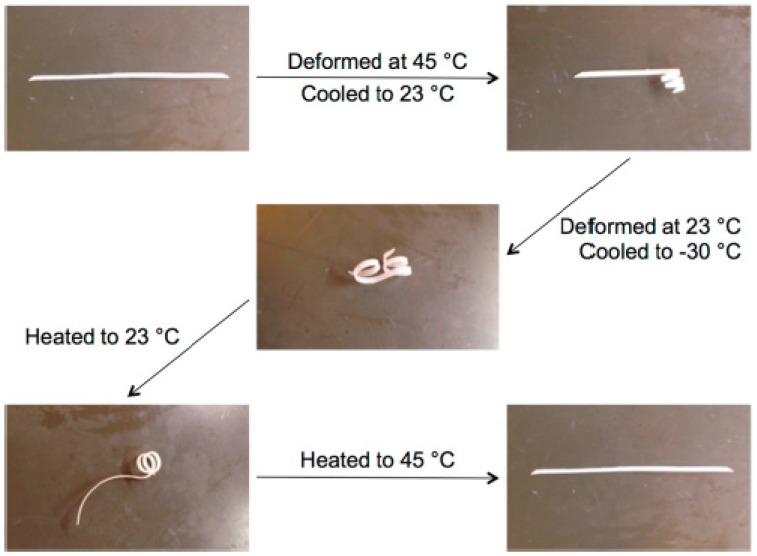
Triple shape memory effect of a dual-curing material based on selective thiol–Michael addition to divinyl sulfones and acrylates with two well-separated glass transitions. Adapted with permission from [64]. Copyright: 2014, American Chemical Society.

Apart from the epoxy based polymers in literature for shape-memory systems [95,96,97], dual-curing systems are being developed over the years using acrylate based monomers. The group led by Bowman [64] developed a well-defined Triple Shape Memory Polymer (TSMP) with well-separated glass transitions and strong interfaces where the thiol−Michael addition reaction was used to form step growth networks based on selective addition to divinyl sulfones and acrylates. The developed TSMP was proved to be stable in its intermediate temporary shape for an extended time thanks to these two distinct networks, each holding separate temporary shapes (Figure 1).

For shape memory devices in biomedical applications, such as stents used in minimally invasive treatment of aneurysms or in suture anchors, a high modulus is required of prepared materials to be able to function effectively [98]. Also, a switching temperature is required to be near physiological temperature. Nair and co-workers [41] developed a dual-cure shape-memory polymer system based on off-stoichiometric thiol–acrylate formulations with a set of intermediate thermal–mechanical properties (*T*_g_ = 33 °C, 20 MPa in modulus and high strain) suitable for deployment of a biomedical device, while the activation of the second stage of the curing process, after deployment, led to a final set of properties (high modulus, 1500 MPa at 38 °C) appropriate for the long-term success and function of the device. 

### 7.2. Optical Materials

Acrylate dual-curing systems have also been evaluated as low refractive index structures. Bowman and co-workers [69] synthesized thiol–isocyanate–acrylate ternary networks formed by the combination of thiol–isocyanate coupling, thiol–Michael addition, and acrylate homopolymerization with excellent optical properties (refractive index about 1.55), enabling extensive use as materials for optical lenses. The same research group obtained similar remarkable refractive index [57] with thiol–isocyanate–ene ternary networks prepared by sequential and simultaneous thiol–ene and thiol–isocyanate click reactions.

### 7.3. Lithography and Photopatterning

Nanoscale lithographic patterns can be manufactured using dual-curing acrylates. Nair et al. [41] used off-stoichiometric thiol–acrylate systems to obtain soft intermediate materials that were used to take the imprint of a micron-size pattern mould and then irradiated to obtain a rigid and highly crosslinked material producing excellent negatives of the master pattern. Whereas the intermediate material had a *T*_g_ of −10 °C and a rubbery modulus (*E*_r_) of 0.5 MPa, the final material had a *T*_g_ of 195 °C and an *E*_r_ of 200 Mpa.

**Figure 2:**
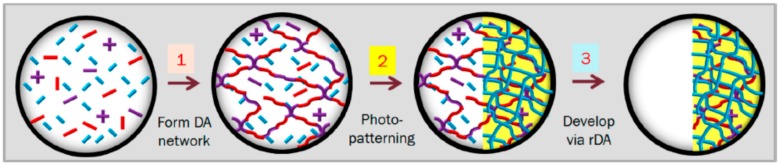
The liquid mixture of monomers is cast as a thin film and heated up to 70 °C, at which temperature the Diels–Alder (DA) network is formed, leaving unreacted monomers. As the monomer conversion exceeds the Flory–Stockmayer threshold for gel point, the film solidifies (Stage 1). Using a photomask, the film is UV irradiated to form the polyacrylate network (Stage 2). When the film is heated to 120 °C the thermoreversible DA network depolymerizes. The resulting liquid monomers can be washed away easily, leaving intact the polyacrylate network (Stage 3). Reprinted with permission from [80]. Copyright: 2014, American Chemical Society.

Using an elegant procedure, Bowman and co-workers [45] successfully implemented different photocaged catalysts in both photopatterning and kinetically controlled two-stage polymer network formation based on thiol–Michael addition to acrylates and methacrylates. They demonstrated the capability of photo thiol–Michael addition for spatial and temporal control. Layer-by-layer photopatterning to produce complex 3D structures was also demonstrated in dual-curable systems combining thermo-reversible Diels–Alder reaction with non-reversible acrylate homopolymerization [80]. The unexposed material could easily be removed by heating to produce depolymerization of the Diels–Alder network followed by washing with a suitable solvent. The whole photolithography process is depicted in Figure 2.

### 7.4. Creating Surface Topology

**Figure 3:**
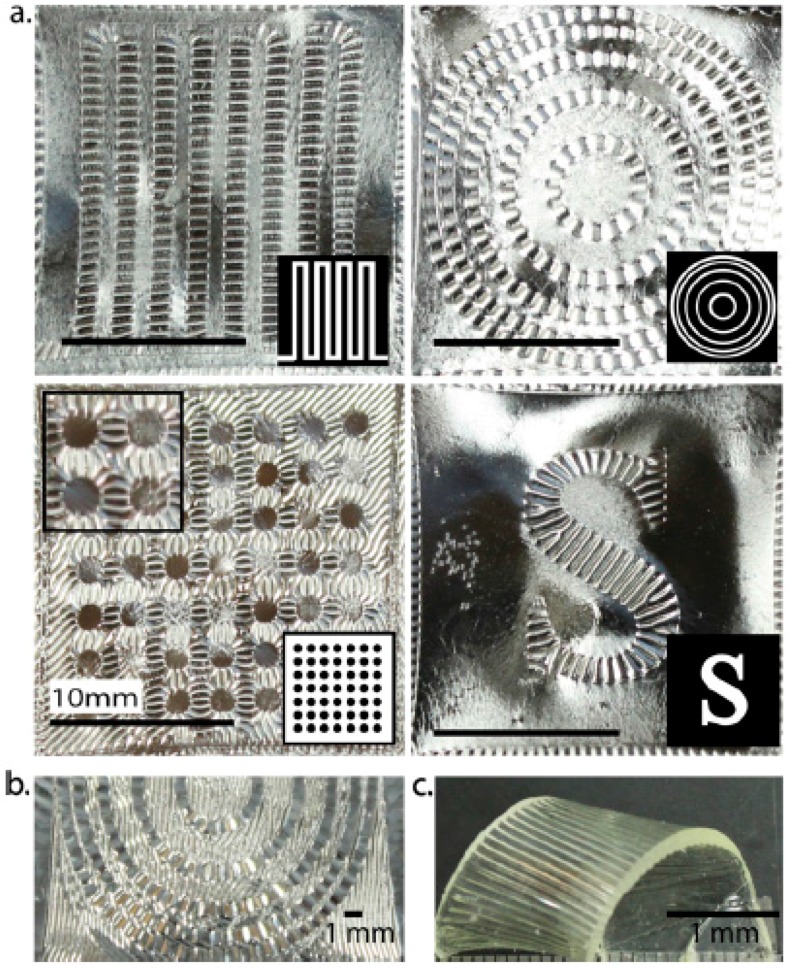
Wrinkle formation via photopatterning on a biaxially stretched specimen. (**a**) Photolithography guides the alignment of the wrinkles perpendicular to the low stress regions of the thiol−ene elastomer (inset: corresponding photomasks). (**b**) Photopolymerized sample showing alternating wrinkle wavelengths, created by stepwise dosing (**c**) Employing two different photoinitiators allows for wrinkle formation and shape memory. Wrinkles were formed using a procedure similar to (**a**). Reprinted (adapted) with permission from [54]. Copyright: 2013, American Chemical Society.

Wrinkled polymeric surfaces having structured topologies with large range of length-scales are efficiently used as coating defence against barnacle fouling [99] or improving adhesive properties of materials [100]. To control wrinkle formation and their orientation, Kloxin and co-workers [54] used a facile two-stage polymerization strategy based on off-stoichiometric thiol–acrylate formulations. Acrylate-rich elastomers that contain photoinitiator and photoabsorber were mechanically stretched and irradiated with UV light. Since the photoabsorber confines the light-induced curing reaction within a thin skin layer at the surface, upon releasing the strain, the resulting cross-link density gradient (i.e., a modulus gradient) causes wrinkle formation (see Figure 3).

To obtain well-defined surface patterns and functional geometric shapes, Bowman’s research group [79] synthesized thiol–isocyanate–methacrylate ternary networks using a dual-curing process. At stage 1, uniform and tough thiourethane networks with a *T*_g_ = 40–70 °C were obtained with a fixed content of fully tethered methacrylate functionalities that served as reactive sites for stage 2 methacrylate UV-induced homopolymerization. The use of suitable photomasks and gradient stage 2 curing and properties across the film thickness made it possible to mechanically program specific 3D shapes or pre-defined surface topographies that could be erased with temperature or fixed by UV flood curing. 

### 7.5. Holography

Holography has evolved rapidly over the years in a number of applications such as art items, data storage, optical elements, artificial intelligence, optical interconnects, commerce, medical practice, night vision goggles and games. However, lack of suitable recording medium is still a bottleneck in this technology. Compared to conventional holographic materials such as dichromated gelatine and silver halide emulsions, photopolymers perform better in recording and reading holograms in real time. Furthermore, these materials possess characteristics such as good light sensitivity, real-time image development, large dynamic range, good optical properties, format flexibility, and low cost. Earlier polymeric systems for holography suffered from low sensitivity due to low curing rates [101] but recently, superior systems are being developed. In the field of non-rewritable holograms, Peng et al. [66] produced rainbow holograms via a sequential orthogonal thiol–click dual curing process. Through an initial thiol–Michael click reaction an intermediate substrate was formed with thiol–allyl photopolymerizable species remaining. Controlling the molar ratio of thiol, acrylate and allyl monomers, intermediate materials with low *T*_g_ and modulus could be prepared to optimize diffusion and reaction rates as required for holographic recording (writing step). After holographic patterning within the crosslinked network was complete, the polymer network was UV irradiated so that the excess thiol and allyl functional groups react, resulting in a material with high modulus, high *T*_g_, and with holographic fringes captured within the substrate. Compared to chain-growth polymerizations, the thiol–ene step-growth reaction exhibits 48% lower volumetric shrinkage, much higher gel point conversions, and better oxygen tolerance. Holograms with high fidelity, good optical clarity and high light sensitivity can be produced without the need for solvent processing and/or protective layers to prevent oxygen inhibition. The same group [67] implemented direct image patterning through the radical-mediated thiol–yne click reaction on self-supporting substrates that were formulated through the base-catalysed thiol–Michael addition followed by a photoinitiated thiol–yne. The holographic patterns attained desirable properties such as high diffraction efficiency, high refractive index modulation, high dynamic range, and low volume shrinkage.

## 8. Conclusions and Future Directions

Acrylates will continue to be employed globally in polymer formulations in different ways. A vast selection of acrylate monomers and oligomers with different backbones and functionalities are commercially available. Their click behaviour has been exploited widely in Michael-type reactions with key coreactants such as thiols or amines. A major challenge of such reactions in dual-curable formulations is the runaway reaction kinetics of Michael reactions. Several researchers have investigated latent catalytic systems in an attempt to address this issue. Consequently, the design of environmentally benign catalytic systems with well-defined curing kinetics stands out as a promising research avenue. Such controlled kinetics will also help in obtaining stable intermediate materials that would cure fully at a later stage, when triggered.

From a physical standpoint, the intermediate materials should provide ease in handling, manipulability and latent reactivity for further curing. This is an interesting research topic that could benefit from multi-disciplinary efforts by organic chemists, reaction engineers, material scientists, and the medical community as these materials also find use in medical settings (such as stents with shape-memory capability). The design of reactive catalysts (the comonomer approach) that impact both the reaction kinetics and rheological/mechanical properties of the final materials (since they get incorporated in the final polymer network) is another research area. 

Last but not the least, more and more research is being directed towards green processes for thermoset preparation. Recently, Acik et al. [102] synthesized acrylates using natural sugar-based monomers. Furthermore, the enzymatic synthesis of photoinitiators have been reported [103]. This avenue of research is particularly important as it could reduce the dependence on non-renewable resources. It is of interest to identify bio-based monomers that can be directly polymerized without need for rigorous purification. For this end, plant oils have been examined thoroughly by Llevot [104] as a bio-based feedstock to synthesize polymers such as polyepoxides, polyurethanes, and unsaturated polyesters. It would be beneficial to investigate the viability of similar approaches to synthesize bio-based polyacrylates.
